# Unveiling embryonal carcinoma: a rare clinico-pathological entity

**DOI:** 10.11604/pamj.2025.52.146.49449

**Published:** 2025-12-08

**Authors:** Kashmira Bhaidkar, Punam Sawarkar

**Affiliations:** 1 Department of Panchakarma, Mahatma Gandhi Ayurved College Hospital and Research Centre, Datta Meghe Institute of Higher Education and Research, Salod (H), Wardha, Maharashtra, India

**Keywords:** Embryonal carcinoma, non-seminomatous germ cell tumor, testicular carcinoma

## Image in medicine

Embryonal carcinoma of the testis is a rare, highly aggressive form of non-seminomatous germ cell tumor that often affects young adult males and has a high tendency to metastasise early. Embryonal carcinoma makes up about 40% of mixed germ cell tumors in testicular cancer, but pure cases are rare, only 1-5% worldwide. In India, testicular cancer incidence is low (0.5 per 100,000), much lower than in Western countries. Testicular germ cell tumors account for 1.7% of cancers in India, though data on embryonal carcinoma remain limited. A 43-year-old male presented to the outpatient department of Mahatma Gandhi Ayurved College Hospital and Research Centre with a history of a progressively enlarging, painful swelling involving the scrotum and penis for the past five months. He also reported foul-smelling discharge along with episodes of recurrent bleeding. On detailed history, it was revealed that the patient had a past medical history of hydrocele diagnosed two years prior, for which surgical intervention was performed. After the procedure, a firm scrotal mass recurred within a few months. Surgical excision was undertaken, and histopathological evaluation established the diagnosis of embryonal carcinoma, an aggressive variant of testicular germ cell malignancy. The patient was subsequently referred to an oncologist, where he was placed on multimodal therapy comprising systemic chemotherapy and adjuvant radiotherapy. Low awareness of embryonal carcinoma and other testicular cancers is concerning, as vague symptoms or painless lumps often delay diagnosis.

**Figure 1:**
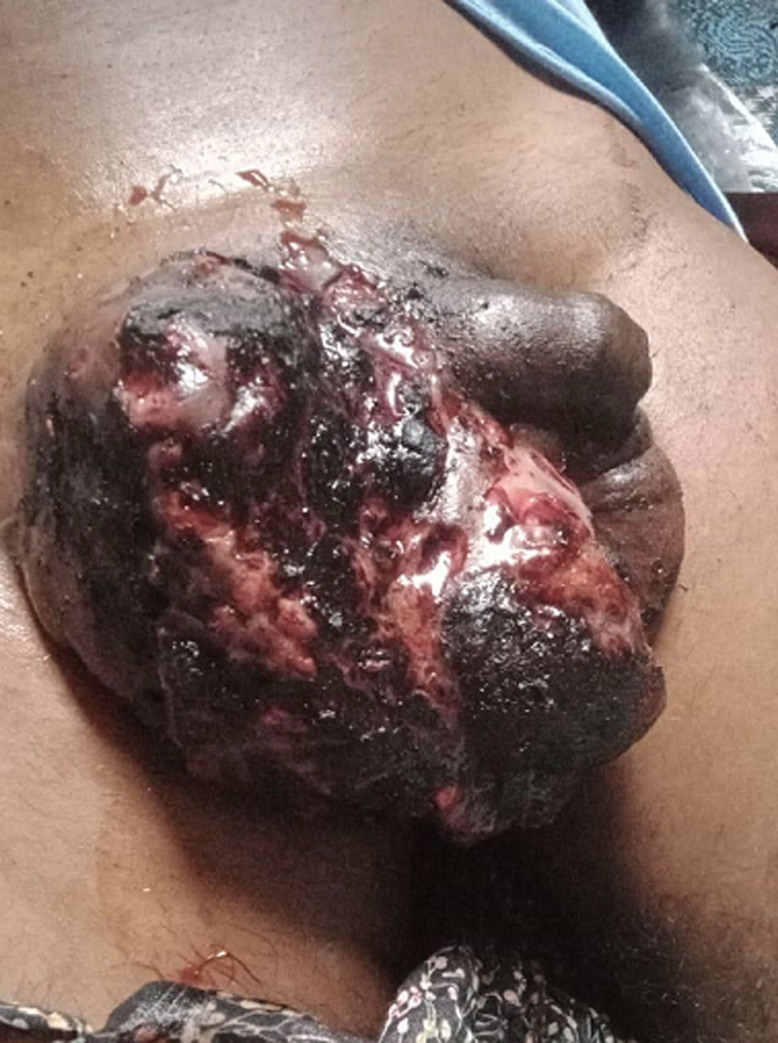
gross appearance of advanced testicular malignancy with fungating scrotal mass

## Available online at

https://www.panafrican-med-journal.com//content/article/52/146/full

